# Uropygial gland size and composition varies according to experimentally modified microbiome in Great tits

**DOI:** 10.1186/1471-2148-14-134

**Published:** 2014-06-17

**Authors:** Staffan Jacob, Anika Immer, Sarah Leclaire, Nathalie Parthuisot, Christine Ducamp, Gilles Espinasse, Philipp Heeb

**Affiliations:** 1 Laboratoire Évolution et Diversité Biologique (EDB), UMR 5174 Centre National de la Recherche Scientifique (CNRS), Ecole Nationale de Formation Agronomique (ENFA) – Université Paul Sabatier, 118 Route de Narbonne, 31062 Toulouse, France; 2 Now at Station d’Ecologie Expérimentale du CNRS à Moulis, USR2936, 09200 Saint-Girons, France; 3 Laboratoire Ecologie et Evolution, UMR 7625, UPMC CNRS ENS, Université Pierre et Marie Curie, 78 quai St Bernard, 75252 Paris, France

**Keywords:** Preen gland, Microorganisms, Host-microbiome interactions, Wax esters, *Parus major*

## Abstract

**Background:**

Parasites exert important selective pressures on host life history traits. In birds, feathers are inhabited by numerous microorganisms, some of them being able to degrade feathers or lead to infections. Preening feathers with secretions of the uropygial gland has been found to act as an antimicrobial defence mechanism, expected to regulate feather microbial communities and thus limit feather abrasion and infections. Here, we used an experimental approach to test whether Great tits (*Parus major*) modify their investment in the uropygial gland in response to differences in environmental microorganisms.

**Results:**

We found that males, but not females, modified the size of their gland when exposed to higher bacterial densities on feathers. We also identified 16 wax esters in the uropygial gland secretions. The relative abundance of some of these esters changed in males and females, while the relative abundance of others changed only in females when exposed to greater bacterial loads on feathers.

**Conclusion:**

Birds live in a bacterial world composed of commensal and pathogenic microorganisms. This study provides the first experimental evidence for modifications of investment in the defensive trait that is the uropygial gland in response to environmental microorganisms in a wild bird.

## Background

Microorganisms such as bacteria and fungi are widespread and constitute the major part of the earth biomass [1,2]. While parasites exert strong selective pressure on host life-history traits [3], beneficial microorganisms can be involved in various processes such as digestion, nutrient synthesis or protection from pathogen colonisation [4-7]. Recently, several studies highlighted the potential role played by the whole assemblage of microorganisms (usually referred as “microbiome” [8]), as selective pressures shaping the evolution of host life history traits [7-12].

Birds carry a large variety of potential pathogens on their plumage [13]. Some can potentially lead to infections [13], while keratinolytic microorganisms have the ability to degrade feathers as found under laboratory conditions [14-16] and might thus alter plumage integrity [16,17]. Alternatively, some microorganisms might be beneficial, for instance by maintaining microbial community stability through competition and cooperation, thus preventing colonisation by environmental pathogens [7,8,18,19]. Given the importance of avoiding pathogen infections and maintaining good plumage integrity, birds are expected to have evolved means to regulate the microorganisms on their feathers [17]. The uropygial gland is an external gland present in almost all bird species, which produces secretions that are coated on feathers during preening. Preen secretions can contain antibacterial substances [20-24]. In the House finch (*Carpodacus mexicanus*), uropygial gland secretions have been found to inhibit the *in vitro* growth of both keratinolytic and non-keratinolytic isolated bacterial strains [20]. Preening feathers with uropygial gland secretions might consequently act as an antimicrobial defence mechanism to regulate microorganisms on feathers [20,21,25,26].

Hosts and their microbiome are involved in reciprocal interactions where a host response can affect its microbial communities [1,2]. Microbial communities present on birds are highly diverse and can show rapid changes in density and composition [27-29]. Consequently, we might expect birds to modify investment in their uropygial gland in relation with the microbial pressures they face [30]. Since uropygial gland secretions and preening behaviour have been suggested to be costly in terms of time, energy and probability of olfactory detection by predators [31,32], we indeed expected birds to adjust gland investment to the levels required for optimal protection against microorganisms [30]. Uropygial gland size and composition of secretions have been found to show seasonal variations and to depend on hormonal levels [32-34]. The uropygial gland is thus a plastic trait that might consequently vary depending on the need for microbial protection. Several studies have examined the effects of uropygial gland secretions on microorganisms [20,25,26,35] and the correlations between gland size and microbial communities on feathers [21]. However, to date no study has examined experimentally whether birds respond to their exposure to environmental microorganisms by modifying their investment in uropygial gland size and/or composition of secretions.

In this study, we experimentally modified Great tit exposure to environmental microorganisms during reproduction to investigate whether birds modify their investment into their uropygial gland in relation to their microbiome. We randomly allocated nests to three treatment groups: two groups of nests were sprayed with liquid solutions that either favoured or inhibited bacterial growth, and a third group acted as control. Since birds are in contact with their nests during breeding, we expected these treatments to affect bacterial communities on bird feathers. We know little about the influences of environmental microorganisms on feather microbial assemblages. Consequently, investigating how modifications of nest microbiome affect the density and composition of feather microbial communities will help to understand the link between environmental bacterial communities and those carried by birds on their feathers.

We measured the volume of the Great tit uropygial gland and analysed the chemical composition of the gland secretions at the end of the reproductive event. We predicted that birds should adjust uropygial gland investment in function of their exposure to microorganisms. Great tits males and females differ in exposure to nest microorganisms, reproductive strategies, immunity and uropygial gland size [29,36-39]. We thus expected that modified bacterial exposure should lead to sex-specific differences in changes of gland volume and composition. Given our limited knowledge of the ecological interactions between microbial communities and hosts, we could not make *a priori* predictions on the direction of the expected effects of treatments on gland investment and whether Great tits adjust the size and/or composition of their secretions. However, effects of modifications of Great tit microbiome would provide the first experimental evidence for a role of the microbial environment in bird investment in the defensive trait that is the uropygial gland.

## Methods

### Experimental design

The study was performed during the reproductive seasons 2011 and 2012 on a Great tit population breeding in nest boxes close to Toulouse, France (43° 39’ N, 1° 54’ E). In the winter, old nest material was removed from the nest boxes and boxes were scraped with a hard brush. Nest boxes were visited daily from the beginning of March to detect the beginning of nest building.

In order to modify bird microbiome, we randomly assigned the nests to three treatments. Firstly to favour the bacterial growth in the nests we used TSB (Tryptic Soy Broth, 40 mg/L in sterilized distilled water, Sigma), a liquid general growth media for heterotrophic microorganisms commonly used in microbiology. Nisin in association with EDTA, a bacteriostatic solution used for food conservation (7 g Nisin (900 IU/mg; B&K Technology Group) *in* 50 mM EDTA [40,41]) was used to inhibit bacterial growth in the nests. TSB and Nisin were diluted in water, and humidity can favour microbial growth [42,43]. Consequently, we used water as a control in order to have similar humidity levels in the three treatments. Differences between treatments in Great tit uropygial gland would thus result from effects of TSB and Nisin solutions on bacterial communities and not from potential humidity effects. After carefully removing the eggs or the nestlings, the three solutions (TSB, Nisin and water) were sprayed (mean volume 1.7 ± 0.02 ml) in the centre of the nest cup every two days during the whole reproductive period (from the beginning of nest building to nestling fledging; total number of treatments per nest; mean ± SE: 16.6 ± 0.3; no significant difference between treatments: ×^2^ = 4.02; df = 52; p = 0.13). During incubation, nests were treated only on day 1, 5 and 9 after the start of incubation in order to limit the risks of nest desertion. A total of 54 nests were included in our study (17 nests in the TSB treatment, 17 in Nisin and 20 in control) and they did not differ significantly in laying date (×^2^ = 3.85; df = 52; p = 0.15) and clutch size (×^2^ = 2.60; df = 52; p = 0.27).

To measure the effects of the treatments on nest bacterial communities, we collected two samples of nest material using sterilized tweezers. Samples were taken from a standardized position in the centre of the nest cup at day 9 of incubation, just before spraying the treatment. One sample was placed in a sterile Eppendorf tube filled with 1 ml Phosphate Buffer Saline (PBS) for DNA extraction, the second into PBS with 20% Glycerol. Glycerol limits crystallization and cellular death when stored at −20°C, and therefore allow us to make culture-based analyses. Samples were kept in ice in the field, and stored at −20°C until lab analyses. All sampling and manipulations were made after systematically washing hands and material with 70% ethanol in order to avoid cross contaminations. All manipulations were performed according to French legislation and permits were obtained from DREAL (Direction Régionale de l’Environnement, de l’Aménagement et du Logement) and CRBPO (Centre de Recherches sur la Biologie des Populations d’Oiseaux; ringing permit N° 565).

### Adult sampling and measurements

Great tits were trapped in the nest boxes around day 10 post hatching (54 females and 44 males), 35.2 ± 0.6 days after the beginning of the treatments. We collected twice 10 feathers samples from each individual at a standardized position close to the left leg. As for nest material samples, one sample was placed in PBS, and the other in PBS + Glycerol. We measured tarsus length to the nearest 0.01 mm using a calliper, body mass with an electronic balance (±0.01 g) and wing length with a ruler (±0.1 mm). We found no significant differences in adult tarsus length, wing length and body mass between the treatments (Tarsus length: F_2,51_ = 1.48; P = 0.24; Wing length: F_2,51_ = 2.06; P = 0.14; Body mass: F_2,51_ = 2.74; P = 0.08).

We measured the length, width and height of the uropygial gland with a calliper (±0.01 mm), each one three times, and multiplied the mean values to obtain an index of uropygial gland volume (L*W*H [44]). Uropygial gland volume was not measured in 2 males, resulting in a sample of 42 males and 54 females in our analyses. In order to avoid any potential observer bias in uropygial gland size measurements, SJ performed all measurements holding the calliper with the scale pointing downward, the values thus visible for the observer only after the measurement. During the second year of the study, we sampled gland secretions by draining the papilla with a glass capillary. We measured the amount of secretions inside the capillary with a calliper (±0.01 mm) to account for quantity of secretions produced at the moment of sampling, and then placed the capillary in glass vials and stored at −20°C until extraction of organic compounds. Using Great tits included in this experimental study and others captured using mist-nets during autumn of the same year, we found that the volume of the gland is positively correlated with the quantity of secretions drained from the papilla (F = 41.09; df = 109; P < 0.001). Moreover, the volume of the gland has been suggested to be a better index of production of secretions by the uropygial gland than the quantity of secretions contained inside the papilla at the time of sampling [21]. We used gland volume and not quantity of secretions in our analyses since we made this measurement during the two years of the study. The same observer (SJ) performed all measurements and sampling. Using 20 birds measured twice, we found high repeatability of the mean uropygial gland volume computed as previously described (r = 0.91; df = 20; P < 0.001).

### Uropygial gland composition analyses

Samples of uropygial gland secretions were diluted in 500 μl of hexane, evaporated, and then diluted in 200 μl of dichloromethane and vortexed for 1 min in order to extract organic chemical compounds. Samples were analysed using Gaz Chromatography – Mass Spectrometry (GC-MS; TSQ Quantum; ThermoScientific, plateform MetaToul), with a migration program as follows: 50°C for 1 min, 10°C/min from 50°C to 300°C and then 10 min at 300°C (see Additional file 1 for details). Blanks were interspersed between each sample. Resulting profiles were analysed using Xcalibur software to generate composition matrices. Since we cannot standardize the quantity of secretions sampled by the GC-MS, we used matrix of intra-individual relative quantity of compounds in all analyses [45]. Compounds that migrated in unidentifiable complexes or that were at very low quantity were not included in the analyses, leading to 16 chemical compounds retained. These compounds were wax esters, lipids ranging from 33 to 37 carbons. 10 of them being formally identified using trans-esterification by base methanolysis (Table 1; see Additional file 1 for details). GE and AI performed respectively GC-MS and profiles analyses blindly to the treatments, and CD performed compound identification.

**Table 1:** Composition of Great tit uropygial gland secretions

	**Compound**	**Formula**	**PC1**	**PC2**	**PC3**
A	Pentadecyl octadecanoate	C33H66O2	0.39	−0.11	**0.54**
B	Hexadecyl 9-octadecenoate	C34H66O2	**−0.88**	−0.17	0.24
C	Hexadecyl octadecanoate	C34H68O2	−0.29	**−0.72**	0.16
D	Nonadecyl hexadecanoate	C35H70O2	**0.55**	−0.14	**0.56**
E	Unidentified	C35H70O2	**0.78**	−0.2	0.06
F	Unidentified	C35H70O2	**0.58**	**−0.51**	−0.09
G	Heptadecyl 9-octadecenoate	C35H70O2	**−0.63**	0.45	0.31
H	Heptadecyl octadecanoate	C35H70O2	**0.91**	0.17	0.27
I	Octadecyl 9-octadecenoate	C36H70O2	**−0.58**	−0.41	−0.48
J	Octadecyl 9-octadecenoate	C36H70O2	**−0.85**	0.07	0.24
K	Octadecyl octadecanoate	C36H72O2	−0.04	0.42	**−0.81**
L	Unidentified	C37H74O2	**0.87**	0.2	0.16
M	Unidentified	C37H74O2	**0.85**	0.07	−0.28
N	Unidentified	C37H74O2	**−0.7**	0.49	0.23
O	Unidentified	C37H74O2	**0.8**	0.03	−0.22
P	Nonadecyl 9-octadecenoate	C37H72O2	−0.34	**−0.79**	−0.1

Factor loadings of the three first principal components summarizing the variance in the chemical composition of uropygial gland secretions in breeding Great tits are shown. The first component represents 45.1% of the original variance, the second 14.9% and the third 12.8% (total 72.8%). Each letter indicates a different compound in the GC-MS profiles. Variables included in each principal component are presented in bold.

### Bacterial analyses

We used respectively culture based and culture independent techniques to measure the density and composition of bacterial communities in the nests and on bird feathers. We sonicated and vortexed bacterial samples to detach microorganisms from nest material and feathers [21,46]. To estimate the densities of bacterial communities, we grew them on tryptic soy agar (TSA), a general medium allowing the growth of heterotrophic bacteria. Keratinolytic bacterial densities were estimated with feather meal agar (FMA), a medium containing only keratin as carbon source [21,46]. Petri dishes were incubated for 3 days for TSA and 14 days for FMA, at 24°C for feather samples and 30°C for nest material samples.

We extracted bacterial DNA using Promega extraction protocol (Promega, Fitchburg, WI, USA) from samples stored in PBS. We use ARISA (Automated Ribosomal Intergenic Spacer Analysis) to measure bacterial community composition [47]. We amplified highly variable regions of the bacterial ribosomal operon, and measured the length of the amplified fragments by sequencing to obtain profiles composed of several peaks (see Additional file 2 for details), each peak corresponding to an operational taxonomic unit (OTU). This method allows to estimate the diversity of bacterial communities, and to compare samples based on their structure (i.e. the presence or absence of the different OTUs, [47]). The peak profiles obtained for bacterial communities were analysed using R software with a standardized automatic method [48] in order to obtain the presence/absence data of OTUs. Briefly, this method consists in two steps, the first one aiming at estimating the best shift value and window size to maximize between samples OTUs profile similarity (in this study shift value = 0.1; window size = 3), the second one allowing to apply these parameters to assemble peaks in OTUs for all samples [48]. We did not detect any contamination of the PBS solution used for sampling since control samples did not contain amplified fragments. Due to technical problems, three samples of nest material were not included in the analyses of bacterial densities (2 from control treatment and 1 from TSB). Moreover, we were unable to extract bacterial DNA from 2 nest samples (2 control samples) and 4 feather samples (2 from control treatment, 1 from Nisin and 1 from TSB). All lab and peak profile analyses were performed blindly to the treatments by SJ.

### Statistical analyses

All analyses were performed using R software (version 2.14.0, R Development Core Team 2008). Analyses of differences in bacterial community structure between treatments were performed using non-parametric multivariate analysis of variance based on permutation tests (Adonis; [49]). For the analyses of treatment effects on nest bacterial density, we used linear models with year and date as covariates, whereas linear mixed models (*lme*, *nlme* R package) with nest as a random factor were performed to analyse the effects of treatments on feather microbial densities. Finally, we used Shannon diversity index to test for differences in bacterial diversity between the treatments.

We used a principal component analysis (PCA) in order to decompose the variance of chemical composition of secretions into independent components [45]. We estimated individual body condition through the regression of body mass on tarsus length (body mass = 4.19 + 5.89 × tarsus length; r^2^ = 0.49; T = 5.48; p < 0.001; [50]). We used linear mixed models to analyse the effect of treatments on the volume and composition of the uropygial gland. Date, year, clutch size, body condition and wing length were included as covariates. Year was not included in the analyses of chemical composition since we obtained data only for the second year of the study. Since we expected differences in antimicrobial strategies between sexes, we included a sex by treatment interaction in all models. Treatment nested in the interaction between nest identity and sex was included as a random factor in order to account for the hierarchical structure of our data. Analyses within each sex were performed using linear models (*lm, stats* R package). Backward selection procedures were applied to remove non-significant factors from the models.

## Results

### Bacterial communities in nests and feathers

From the 52 nest samples analyzed for bacterial community composition, we identified 180 OTUs, whereas feather bacterial communities appeared less diverse with 138 OTUs extracted from the 94 feather samples. Nest communities showed 8 OTUs (4.4%) with more than 20% prevalence (max 26%), and 84 OTUs (46.7%) with very low prevalence (<5%). Feather communities are composed of 28 OTUs (20.3%) with more than 20% prevalence (max 67%), and 44 OTUs (31.9%) with very low prevalence (<5%). Finally, the number of OTUs detected averaged 45.06 ± 4.31 in nest samples and 24.87 ± 2.20 in feather samples.

**Figure 1:**
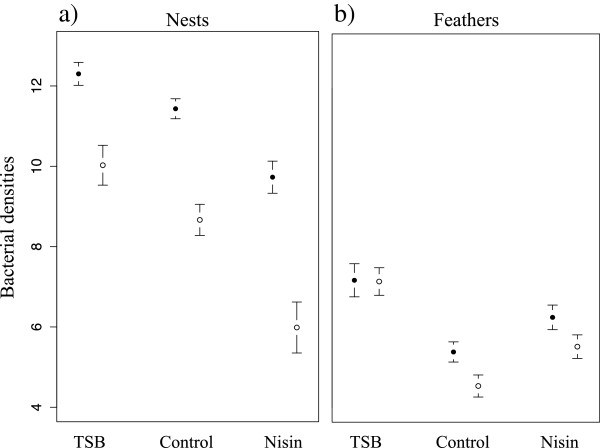
**Experimental modification of bacterial densities in nests and on feathers.** Full circles represent the total cultivable bacterial densities, empty circles the keratinolytic bacterial densities (**a**: nests; **b**: plumage; mean ± SE of log transformed CFU). Bacterial densities on adults from the two sexes were not differently affected by the treatments (treatment * sex interactions, all P > 0.05).

We found a significant effect of the treatments on nest total and keratinolytic bacterial densities (df = 49; F = 16.7; p < 0.001 and df = 49; F = 15.7; p < 0.001 respectively; Figure 1). The TSB treatment significantly increased the total and keratinolytic bacterial densities in the nests compared to the control (Table 2). The Nisin treatment significantly decreased both total and keratinolytic bacterial densities in the nests (Table 2). The structure of the bacterial communities in the nests was significantly affected by the treatments (Adonis analysis, Bray-Curtis distance; df = 49; R^2^ = 0.10; p = 0.007), with a significant difference between TSB and control treatments (Table 2), whereas Nisin had no significant effect on bacterial community structure compared to the control treatment (Table 2). Using Shannon diversity index, we found a significant effect of the treatments on nest bacterial diversity (df = 49; F = 3.16; p = 0.05), with TSB treatment increasing nest bacterial diversity (Table 2) whereas Nisin did not affect nest bacterial diversity (Table 2).

**Table 2:** Effects of treatments on density, diversity and composition of nest and feather bacterial communities compared to the control

	**Total bacterial densities**				**Keratinolytic bacterial densities**			
**Nests**	**Estimate ± SE**	**Df**	**T**	**P**	**Estimate ± SE**	**Df**	**T**	**P**
TSB	0.87 ± 0.38	32	2.30	0.028*	1.36 ± 0.62	32	2.19	0.036*
Nisin	−1.70 ± 0.46	33	−3.67	<0.001***	−2.68 ± 0.73	33	−3.65	<0.001***
**Feathers**	**Estimate ± SE**	**Df**	**T**	**P**	**Estimate ± SE**	**Df**	**T**	**P**
TSB	1.64 ± 0.61	36	2.69	0.011*	2.53 ± 0.52	37	4.90	<0.001***
Nisin	0.87 ± 0.39	34	2.20	0.035*	0.99 ± 0.41	35	2.43	0.021*
								
	**Bacterial diversity**				**Bacterial composition**			
**Nests**	**Estimate ± SE**	**Df**	**T**	**P**	**SumSq**	**Df**	**F**	**P**
TSB	0.60 ± 0.28	30	2.14	0.040*	0.80	30	3.59	0.005**
Nisin	−0.01 ± 0.29	30	−0.02	0.981	0.16	30	0.65	0.715
**Feathers**	**Estimate ± SE**	**Df**	**T**	**P**	**SumSq**	**Df**	**F**	**P**
TSB	0.41 ± 0.25	37	1.62	0.113	1.17	37	7.26	0.002**
Nisin	−0.07 ± 0.32	35	−0.23	0.82	0.08	35	0.51	0.767

Significant effects are annotated by *p<0.05, **p<0.01, ***p<0.001.

The modifications of nest microbiome induced by the treatments (Figure 1) also affected the microbial communities on bird feathers. We found significant differences in total and keratinolytic bacterial loads on feathers between treatments (F_2,52_ = 4.95; p = 0.011 and F_2,53_ = 13.21; p < 0.001 respectively; Figure 1). TSB increased significantly both total and keratinolytic bacterial loads (Table 2). Interestingly, compared to the control, the Nisin treatment significantly increased feather loads of both total and keratinolytic bacteria (Table 2). The feather bacterial community structure significantly differed between the treatments (df = 53; R^2^ = 0.11; p = 0.001), with TSB treatment significantly affecting bacterial community structure on feathers (Table 2). In contrast, Nisin treatment did not significantly affect feather bacterial community structure (Table 2). Analyses of Shannon diversity index showed no significant effect of the treatments on bacterial diversity (Table 2). Bacterial communities on adults from the two sexes were not differently affected by the treatments (treatment * sex interactions, all p > 0.05). However, females carried higher total and keratinolytic bacterial loads (0.70 ± 0.31 (Estimate ± SE), df = 33, T = 2.23, P = 0.033 and 0.79 ± 0.26; df = 36, T = 3.03, P = 0.004 respectively), showed higher bacterial diversity (1.02 ± 0.18, df = 37; T = 5.65, P < 0.001) and different bacterial community composition (df = 53; R^2^ = 0.14; P = 0.001) on feathers compared to males.

### Uropygial gland volume and composition

**Figure 2:**
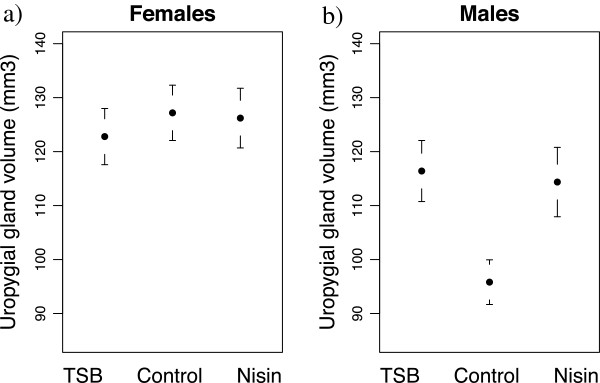
Effects of experimental modifications of Great tit microbiome on uropygial gland volume.

We found a significant interaction between sex and treatment on the volume of the uropygial gland (Table 3a). Uropygial gland volume significantly differed between treatments in males (Table 4a), being bigger in the TSB (0.19 ± 0.07 (Estimate ± SE); F_1,28_ = 8.15; p = 0.008) and Nisin (0.17 ± 0.07; F_1,25_ = 5.30; p = 0.03) treatments compared to the control (Figure 2b). Females had larger uropygial glands than males (0.15 ± 0.04; df = 39; T = 4.34; p < 0.001), but the size of their glands did not differ significantly between treatments (Table 4a, Figure 2a).

**Table 3:** Effects of modification of bird microbiome on uropygial gland volume and composition of secretions

**a**	**Uropygial gland volume**					
			**Estimate ± SE**	**df**	**F**	**P**
	Year		0.20 ± 0.04	1,52	29.6	<0.001
	Wing length		0.53 ± 0.12	1,36	19.6	<0.001
	Treatment	Nisin	−0.05 ± 0.06	2,52	1.2	0.31
		TSB	−0.09 ± 0.06			
	Sex		0.49 ± 0.08	1,36	41.2	<0.001
	Treatment × Sex	Nisin	0.20 ± 0.09	2,36	4.2	0.018
		TSB	0.24 ± 0.09			
**b**	**Uropygial gland composition - PC1**					
			**Estimate ± SE**	**df**	**F**	**P**
	Treatment	Nisin	0.04 ± 0.14	2,28	3.7	0.033
		TSB	−0.30 ± 0.15			
	Sex		0.72 ± 0.12	1,22	38.6	<0.001
**c**	**Uropygial gland composition - PC2**					
			**Estimate ± SE**	**df**	**F**	**P**
	Date		0.05 ± 0.02	1,19	5.7	0.021
	Treatment	Nisin	2.29 ± 0.58	2,28	8.8	<0.001
		TSB	1.97 ± 0.60			
	Sex		−2.89 ± 0.69	1,19	17.3	<0.001
	Treatment × Sex	Nisin	2.36 ± 0.91	2,19	5.2	0.01
		TSB	2.81 ± 0.92			
**d**	**Uropygial gland composition - PC3**					
			**Estimate ± SE**	**df**	**F**	**P**
	Sex		−1.34 ± 0.35	1,22	39.5	<0.001

The compounds produced and their contribution to the principal components are given in Table 1. The table present final models after backward selection. Treatment effects estimates compared to the control are shown.

**Table 4:** Analyses of preen gland volume and PC2 composition of secretions for each sex

**a**	**Uropygial gland volume**						
	**Females**			**Estimate ± SE**	**df**	**F**	**P**
		Date		0.01 ± 0.01	1,50	4.25	0.04
		Year		0.15 ± 0.04	1,50	13.89	<0.001
		Wing length		0.41 ± 0.13	1,50	10.6	0.002
	**Males**			**Estimate ± SE**	**df**	**F**	**P**
		Date		−0.01 ± 0.004	1,36	7,0	0.01
		Year		0.21 ± 0.06	1,36	13,0	0.001
		Body condition		0.16 ± 0.04	1,36	12.5	0.001
		Treatment	Nisin	0.16 ± 0.07	2,36	4.3	0.02
			TSB	0.16 ± 0.06			
**b**	**Uropygial gland composition - PC2**						
	**Females**			**Estimate ± SE**	**df**	**F**	**P**
		Date		0.07 ± 0.03	1,27	5.5	0.03
		Treatment	Nisin	2.38 ± 0.69	2,27	6.5	0.005
			TSB	1.95 ± 0.71			
	**Males**			**Estimate ± SE**	**df**	**F**	**P**
		Body condition		−0.51 ± 0.22	1,21	5.4	0.03

The compounds produced and their contribution to the principal components are given in Table 1. The table present final models after backward selection. Treatment effects estimates compared to the control are shown.

Using Shannon diversity index, we found that the mean diversity of chemical compounds contained in gland secretions did not differ significantly between males and females (0.03 ± 0.04; F_1,21_ = 0.69; p = 0.41). A principal component analysis summarizing the chemical compounds in the gland secretions revealed that a first principal component stands for variation in 12 of the 16 components (7 positively and 5 negatively; Table 1) and represent 45.1% of the original variance. The second principal component stands for 14.9% of the variance and positively correlates with 3 compounds whereas the third represent 12.8% of the variance and correlates with 3 compounds (Table 1).

**Figure 3:**
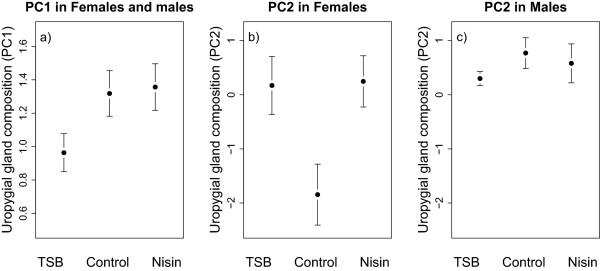
**Effects of experimental modifications of Great tit microbiome on the composition of uropygial gland secretion.** PC1 in both sexes **(a)**, PC2 in females **(b)** and PC2 in males **(c)** were presented. The compounds produced and their contributions to the principal components are given in Table 1.

We found a significant effect of the treatments on the compounds forming the first principal component (PC1) of uropygial gland secretions (Table 3b; Figure 3a), with an almost significant difference between TSB and control treatments (0.32 ± 0.15; F_1,17_ = 4.33; p = 0.053), but not between Nisin and control (0.04 ± 0.13; F_1,19_ = 0.08; p = 0.77). The compounds forming the first and third components (PC1 and PC3) of the uropygial gland secretions differed significantly between males and females (Table 3b, d). Moreover, we found a significant interaction between treatment and sex on the scores of the second component (PC2; Table 3c). Separate analyses within each sex revealed a significant effect of the treatments on the second component (PC2) of females but not of males (Table 4b; Figure 3b, c), with females of both TSB and Nisin treatments that significantly differed from control (2.02 ± 0.78; F_1,17_ = 6.74; p = 0.019 and 2.09 ± 0.73; F_1,19_ = 8.16; p = 0.010 respectively). These effects are not related to investment in reproduction since we found no significant effects of clutch size in our analyses (Table 3, 3).

## Discussion

The experimental design we used in this study consisted in favouring or inhibiting microorganisms in the nests of Great tits using a general growth media (TSB) or a large spectrum bacteriostatic (Nisin). Since Great tits are in contact with nest materials during reproduction, we expected these treatments to affect feather bacterial communities. Moreover, since uropygial gland secretions have been found to regulate feather microbial communities [20-24], we expected birds to adjust the volume and composition of this anti-microbial gland depending on their exposure to microorganisms. Accordingly, we found that modifications of Great tit microbiome led to changes in uropygial gland size and composition, providing experimental evidence for microbial induced changes in investment in the uropygial gland.

As expected since Great tits are exposed to nest microorganisms during breeding [29,51], we found that the modifications of nest bacterial communities affected feather communities. We found higher bacterial loads on feathers of females than males. Interestingly, we found no significant interaction between treatment and sex on feather bacterial communities, showing that treatments effects on feather bacteria did not significantly differ between the sexes. This result suggests that although females spend more time than males in the nests for nest building, egg laying, incubation and nestling raising [39], feather bacterial communities of both sexes were equally affected by nest microorganisms.

Interestingly, whereas TSB led to an increase of cultivable bacterial densities both in the nests and on adult feathers, Nisin led to a decrease of bacterial densities in the nests, but not on feathers. Instead, it led to higher loads of both total and keratinolytic bacteria on feathers compared to controls. However, bacterial loads on feathers were lower than densities found in the nests. Competition among microorganisms for space and resources within the microbiome can prevent colonisation by environmental microorganisms [7,8,18]. Inhibiting bacterial growth in Great tit nests by the Nisin treatment might have destabilized the feather bacterial community, decreasing the ability of certain microorganisms to outcompete others. Moreover, since birds have been found to regulate their feather bacterial communities by using secretions from their uropygial gland [20,21], the bacterial densities observed on bird feathers might result partly from the ability of birds to shape feather microorganisms using their uropygial gland [20,21,25,26]. Disentangling the effects of nest and feather bacterial communities on bird phenotypic traits and investigating the factors that shape feather bacterial communities represent promising and important steps for future research in bird-microorganisms interactions.

We found that both sexes modified the production of certain wax esters in gland secretions (summarized in PC1) in response to the TSB treatment. Moreover, we found that only males increased the volume of their uropygial gland in the TSB and Nisin treatments (i.e. when bacterial loads were increased on the plumage) compared to the control. Variation in the quantity of secretions produced by birds has been previously correlated to feather mite and bacterial densities [21,44,52], and secretions have been found to inhibit the growth of five isolated bacteria *in vitro*[20]. Moreover, Hoopoes (*Upupa epops*) harbour symbiotic bacteria that produce antimicrobial substances in their uropygial gland, thus protecting them against potential pathogenic microorganisms [19,26,53-55]. In our study, the increase in uropygial gland volume in males exposed to higher bacterial loads on feathers reveal that male Great tits increased their overall investment in the uropygial gland when facing high bacterial exposure.

In contrast to males, females did not significantly modify the volume of their gland according to their microbiome, but modified some wax esters (summarized in PC2) in their secretions when exposed to higher bacterial densities on their feathers. As found in many bird species [45,56-58], the uropygial gland secretions in Great tits are composed of wax esters here ranging from 33 to 37 carbons. Wax esters, as all lipids, are energy stores that can be used by some microorganisms for growth [59,60]. Therefore, by preening feathers with wax esters, birds might favour the growth of commensal or mutualistic microorganisms on feathers and as a result limit the colonisation or activity of pathogenic microorganisms through competition between microorganisms [5,6,19,61]. However, whether the changes of gland size and composition of secretions led to adaptive regulation of feather bacteria require further experiments. Testing the consequences of changes in quantity and composition of the uropygial gland secretions for regulation of feather bacterial communities is thus an important path for future research about bird-microorganisms interactions.

Three different characteristics of the uropygial gland (i.e. volume, secretion components in PC1 and PC2) varied according to the bacterial modifications in our study. We found that the TSB and Nisin treatments led to modifications of uropygial gland volume in males, and secretion components summarized in PC2 in females. In contrast, the components in PC1 changed in the two sexes only in response to the TSB treatment. Our results thus showed that the multiple components of the uropygial gland were differentially affected by changes in bird exposure to microorganisms. Some compounds (summarized in PC1) might vary in response to changes in the bird microbiome composition, since only the TSB treatment led to significant changes in nest and feather microbiome composition and also affected compounds in PC1. In contrast, the volume in males and certain secretion components in females (summarized in PC2) might vary according to the bacterial densities on bird feathers, since both treatments led to an increase of the bacterial densities on feathers. In this study, although nest bacterial densities were higher than feather densities, our results suggest that birds did not adjust investment in their uropygial gland according to nest microorganisms, but to those present on their feathers. Our results are in accordance with previous studies suggesting that one main function of the uropygial gland is to regulate feather microorganisms [20,21,25].

Importantly, here we sprayed water in the nests as a control in order to mimic the increase in dampness induced by the TSB and Nisin treatments. Since humidity has been found to affect microbial growth, our control treatment might also have modified the Great tit microbiome, for instance by having favoured bacterial growth in the nests. As a consequence, bacterial densities in our control treatment might be higher than natural bacterial densities in the absence of any manipulation. However, this control treatment was adapted to our experimental design that aimed at testing for differences in Great tit uropygial gland investment between different levels of bacterial exposure. Although additional studies investigating uropygial gland size and composition in relation to natural unaltered microbiome will certainly provide important knowledge about bird-microorganisms interactions, our experimental study showed that Great tits exposed to different levels of bacterial densities modified their investment in their uropygial gland.

The effects of bird microbiome modifications on investment in uropygial gland found here might result from various proximate mechanisms such as skin infections or ingestion of microorganisms [62]. For instance, birds are known to ingest microorganisms present on their feathers during preening [62]. Consequently, the increased bacterial loads on feathers in the TSB and Nisin treatment might result in an increased ingestion of microorganisms, leading to modifications of uropygial gland investment. Recently, it has been found that an increase of testosterone levels stimulates the production of four volatile compounds in captive Dark-eyed juncos (*Junco hyemalis*) [34]), showing that hormonal regulation can affect the production of chemical compounds contained by the uropygial gland. However, further experiments are required in order to identify the proximate mechanisms responsible for variations in uropygial gland investment in response to microbial exposure.

## Conclusions

Birds live in a bacterial world composed of commensal and pathogenic microorganisms [1,2,13,17]. Some of these microorganisms can degrade feathers [17], modify predation risks [63], or, after been ingested, affect gut microbial communities and potentially health [62,64,65]. The uropygial gland function as a defence mechanism to avoid colonisation and maintenance of pathogenic microorganisms on feathers, and thus protect birds from infections and feather degradation [20,21,25,26]. Here we showed, for the first time, that modifications of Great tit microbiome affected investment in the quantity and composition of uropygial gland secretions. Future studies should examine the respective role of quantity and composition of uropygial gland secretions on the regulation of the various pathogenic or beneficial microorganisms inhabiting bird feathers.

## Availability of supporting data

The data sets supporting the results of this article are available in the Dryad repository doi:10.5061/dryad.m8d1c [66] (http://doi.org/doi:10.5061/dryad.m8d1c).

## Competing interests

The authors declare that they have no competing interests.

## Authors’ contributions

SJ and PH defined the research theme, designed methods and performed the experiment. SJ and NP performed the cultured-based analyses, SJ the cultured-independent analyses, SL, GE and CD the chemical analyses, and SJ and AI analysed the data from chemical analyses. SJ achieved the statistical analyses and wrote the manuscript. All authors contributed to the manuscript writing and approved its final version.

## Supplementary Material

Additional file 1 Overview of the chemical identification of compounds contained in Great tit uropygial gland. Click here for file

Additional file 2 Details on culture-independent microbial analyses and chemical analyses. Click here for file
