# Supplementary material for: Uropygial gland size and composition varies according to experimentally modified microbiome in Great tits
Source: BMC Evol Biol. 2014 Jun 17;14:134. doi: 10.1186/1471-2148-14-134 (PMC4074404; doi:10.1186/1471-2148-14-134)
Supplement: Additional file 1 — Overview of the chemical identification of compounds contained in Great tit uropygial gland. [file 1471-2148-14-134-S1.docx]

**SUPPLEMENTARY MATERIAL**

**Culture-independent microbial analyses**

We obtained microbial suspensions from samples stored in PBS using the same sonication protocol than for PBS + Glycerol samples. Then we extracted bacterial DNA using Promega extraction protocol (Promega, Fitchburg, WI, USA). We analysed the structure and diversity of the bacterial communities using automated ribosomal intergenic spacer analysis (ARISA [1]. This DNA-fingerprinting method consists in amplifying the internal transcribe spacer (ITS) region between the 16S and 23S rRNA genes in the ribosomal operon. Given that this region is highly variable between bacterial species, analysing the length of the amplified fragments using sequencing allow obtaining profiles composed of several peaks, each peak corresponding to an operational taxonomic unit (OTU). Consequently, this method allows to estimate the diversity of bacterial communities, and to compare samples based on their structure (i.e. the presence or absence of the different OTUs [1]).

The 16S – 23S region was amplified using the S-D-Bact-1522-b-S-20 and L-D-Bact-132-a-A-18 primers [1]. The PCR reaction was conducted in 20 μl with 200 μM dNTP, 0.25 μM of each primer, 2 U of Taq polymerase and the corresponding Taq buffer (Qiagen), and 2 μl of extracted DNA. Amplification program used was as following: initial denaturation at 94°C for 3min, 5 cycles at 94°C for 45s, 60°C for 45s and 72°C for 1min, followed by 30 cycles at 94°C for 45s, 55°C for 45s and 72°C for 1min, and finally 72°C for 10min. PCR products were then mixed with 9 μl HiDi formamide and 0.1 μl 1200LIZ size standard. The solutions mixtures were denaturized at 95°C for 5 min before separation with a capillary sequencer ABI 3730 (Applied Biosystem).

# Chemical analyses

The GC-MS analyses were performed on a GC-MS TSQ Quantum (ThermoScientific, plateform MetaToul). The temperature source was set at 200°C, the interface between GC and MS modules at 250°C and the splitless injector at 280°C. Helium was the carrier gas and the flow rate was 1.2 ml/min. Samples of 1 µL were injected in an apolar capillary column (5MS 30 m x 0.25 mm, 0.25 µm film thickness, 5% diphenyl and 95% dimethylpolysiloxane). The chromatograph oven was programmed as follows: 50°C for 1 min, then from 50 to 300°C at 10°C per min, and finally held at 300°C for 10 min. The mass spectra were scanned from 60 to 500 m/z. The whole system was controlled by Xcalibur data system, 2.1 version. Detection limits of chromatographic peaks were automatically established by the Xcalibur software.

Esters were trans-esterified [2]. The samples were treated with a solution of KOH in dry methanol (0.5M; 25µL) and the mixture was held at room temperature for 30 min. The mixture was neutralized with dilute HCl, and then extracted with hexane. The samples were kept at 4°C until analysed using GC-MS. Finally, we identified compound’s structure using the mass spectral fragmentation patterns, comparison of m/z ratios with known compounds [3] and NIST library spectra.

**REFERENCES**

1. Ranjard L, Brothier E, Nazaret S (2000) Sequencing bands of ribosomal intergenic spacer analysis fingerprints for characterization and microscale distribution of soil bacterium populations responding to mercury spiking. Appl Environ Microbiol 66: 5334–5339.

2. Haynes K, Millar J (1998) Methods in Chemical Ecology Volume I· Chemical Methods. Springer.

3. Patel S, Nelson DR, Gibbs a G (2001) Chemical and physical analyses of wax ester properties. J insect Sci: 7pp. Available: http://www.pubmedcentral.nih.gov/articlerender.fcgi?artid=355888&tool=pmcentrez&rendertype=abstract.
